# LightRoseTTA: High‐Efficient and Accurate Protein Structure Prediction Using a Light‐Weight Deep Graph Model

**DOI:** 10.1002/advs.202309051

**Published:** 2025-03-25

**Authors:** Xudong Wang, Tong Zhang, Guangbu Liu, Zhen Cui, Zhiyong Zeng, Cheng Long, Wenming Zheng, Jian Yang

**Affiliations:** ^1^ School of Computer Science and Engineering Nanjing University of Science and Technology Nanjing 210094 China; ^2^ School of Automation Nanjing University of Science and Technology Nanjing 210094 China; ^3^ School of Computer Engineering Nanyang Technological University No. 50, Nanyang Avenue Singapore 639798 Singapore; ^4^ School of Biological Science & Medical Engineering Southeast University Nanjing 210096 China

**Keywords:** graph neural network, light‐weight deep learning model, protein structure prediction

## Abstract

Accurately predicting protein structure, from sequences to 3D structures, is of great significance in biological research. To tackle this issue, a representative deep big model, RoseTTAFold, is proposed with promising success. Here, “a light‐weight deep graph network, named LightRoseTTA,” is reported to achieve accurate and highly efficient prediction for proteins. Notably, three highlights are possessed by LightRoseTTA: i) high‐accurate structure prediction for proteins, being “competitive with RoseTTAFold” on multiple popular datasets including CASP14 and CAMEO; ii) high‐efficient training and inference with a light‐weight model, costing “only 1 week on one single NVIDIA 3090 GPU for model‐training” (vs 30 days on 8 NVIDIA V100 GPUs for RoseTTAFold) and containing “only 1.4M parameters” (vs 130M in RoseTTAFold); iii) low dependency on multi‐sequence alignment (MSA), achieving the best performance on three MSA‐insufficient datasets: Orphan, De novo, and Orphan25. Besides, LightRoseTTA is “transferable” from general proteins to antibody data, as verified in the experiments. The time and resource costs of LightRoseTTA and RoseTTAFold are further discussed to demonstrate the feasibility of light‐weight models for protein structure prediction, which may be crucial in resource‐limited research for universities and academic institutions. The code and model are released to speed biological research (https://github.com/psp3dcg/LightRoseTTA).

## Graphical abstract

Accurately predicting protein structure is of great significance in biological research. LightRoseTTA, a light‐weight deep graph network, to achieve prediction for proteins is presented. Notably, three highlights are possessed by LightRoseTTA: i) high‐accurate structure prediction for proteins; ii) high‐efficient training and inference; and iii) low dependency on multi‐sequence alignment (MSA). Finally, LightRoseTTA is evaluated on several benchmarks and outperforms alternative methods.

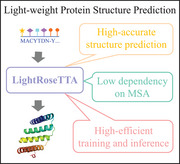

## 1 Introduction

Proteins play significant roles in the biological activities of living organisms, including transporting substances, participating in immunity, and regulating hormones. As the function of proteins is largely determined by the structure, making clear 3D structures would give important insights into understanding biological mechanisms in cellular processes and facilitate interventions (e.g., drug development) through protein function modulation. The previous conventional methods of experimental structure determination, using either nuclear magnetic resonance,^[^ ^1^ ^]^ X‐ray crystallography,^[^ ^2^ ^]^ or cryo‐electron microscopy,^[^ ^3^ ^]^ can be rather time‐consuming and expensive. Hence, it has become a hot research topic which is to leverage machine learning algorithms to predict 3D protein tertiary structures from amino acid sequences.

Tremendous efforts have been made for protein structure prediction in the past decade. In the early stage, one main technical line primarily considers physical chemistry principles of proteins in structure prediction, e.g., assuming the lowest‐energy conformation for folded proteins. However, it suffers from the difficulties of determining a widely applicable energy function as well as the exhausting search in the huge conformational space. As a result, limited prediction performances were achieved. In recent years, with the ever‐growing sequences in protein databases such as UniProt Knowledge base,^[^ ^4^, ^5^ ^]^ deep learning algorithms have been introduced for protein structure prediction.^[^ ^6^, ^7^, ^8^, ^9^, ^10^, ^11^ ^]^ Generally, the main‐stream deep models utilize information from both template structures and homologous sequences, and further design neural networks with rather deep architecture to learn the co‐evolved features. Following this pipeline, RoseTTAFold,^[^ ^12^ ^]^ as one of the representative models, achieves promising performance on the CASP14 dataset. However, the deep models heavily depend on multi‐sequence alignment (MSA), and thus obtain poor prediction accuracy on those proteins with few or limited homologous sequences. To solve this issue, some MSA‐free deep learning methods,^[^ ^13^, ^14^ ^]^ named trRosettaX‐single and RGN2, have been proposed to predict structures by using only textual context of amino acid sequences without MSA, effectively improving the performance on the proteins with few homologous sequences.

Although significant success has been made by previous works, two critical issues still remain in promoting research and application of protein structure prediction. The deep big models suffer from high computational cost and resource burden. For instance, RoseTTAFold needs 30 days to be trained on 8 NVIDIA V100 GPUs. This limits the updating efficiency of the research and model development, meanwhile, deters most universities and academy institutions from participating in this research field. Second, a better MSA‐dependency reduction strategy is urgently required to jointly perform well on both sufficient and insufficient homologous sequences. Although the existing MSA‐free deep learning methods improve the prediction performance on proteins with insufficient homologous sequences, their results on other homology‐sufficient proteins are not satisfying, finally leading to relatively low overall performance.

In this work, we describe a novel light‐weight deep framework, named LightRoseTTA to accurately and efficiently predict 3D all‐atom coordinates of proteins. Primarily, compared with RoseTTAFold, three outstanding advantages are possessed by our LightRoseTTA, i) more competitive performance on protein structure prediction; ii) the much smaller model size with only 1.4M parameters, costing much less computational time and resource in the model‐training stage; iii) the lower dependency on homologous sequences. For the model design, a backbone‐to‐all‐atom architecture is proposed to accurately predict 3D all‐atom coordinates by leveraging high backbone‐sidechain dependency. Specifically, for the backbone conformation, a two‐branch network, taking MSA, template, and atom‐level information as inputs, is designed to model both inter‐residue relationships and sidechain influences. Most critically, to compress the model while achieving high performance, we observe and leverage a constraint prior named backbone potential energy (BPE), normalizing bond lengths, bond angles and dihedrals, to guide the backbone generation. The BPE constraint effectively regularizes the parameter optimization space for backbone generation, enabling a significantly shallower architecture network to handle the complicated backbone conformation as validated in experiments. Moreover, to well handle proteins with both sufficient and insufficient homologous sequences, a new MSA dependency‐reducing strategy is further designed during the model training.

We report the performance of the popular CASP14 dataset (critical assessment of techniques for protein structure prediction^[^ ^15^ ^]^) and CAMEO dataset (continuous automated model evaluation^[^ ^16^ ^]^), as well as the homology‐insufficient Orphan,^[^ ^14^ ^]^ De novo,^[^ ^14^ ^]^ Orphan25,^[^ ^13^ ^]^ and Design55^[^ ^13^ ^]^ datasets. The experimental results demonstrate that more competitive performances (TM‐score^[^ ^17^ ^]^ and GDT_TS^[^ ^18^ ^]^) of our light‐weight LightRoseTTA compared with RoseTTAFold on CASP14 and CAMEO, and homolog‐insufficient datasets Orphan, De novo, and Orphan25. Furthermore, to check the feasibility of LightRoseTTA across different protein categories, e.g., general protein versus antibody, we transfer our LightRoseTTA to predict the antibody structure on the Rosetta Antibody Benchmark.^[^ ^19^ ^]^ LightRoseTTA achieves promising performance on the most critical region–the third complementary determining region ring of the heavy chain (CDR‐H3) for antibodies, verifying its effectiveness and good transferability for proteins of different categories. Moreover, we discuss the time and resource costs of LightRoseTTA and RoseTTAFold for protein structure prediction. Our LightRoseTTA has only 1.4M parameters, and can be well trained on a single GPU (NVIDIA RTX 3090) in only one week. In contrast, RoseTTAFold costs 30 days using 8 NVIDIA V100 GPUs. Our LightRoseTTA achieves the best performances in the most cases while costing far less time and resources for model learning.

## 2 Results

### 2.1 Approach Summary

**Figure 1:**
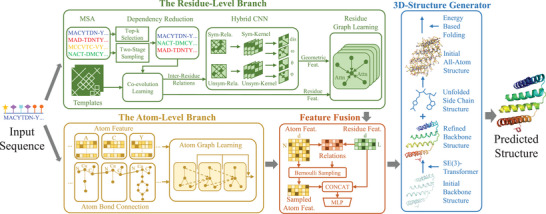
The architecture of LightRoseTTA for protein structure prediction. LightRoseTTA consists of two branches, the residue‐level branch and the atom‐level one. For one given amino acid sequence, three aspects of information, i.e., MSA features, structure templates, and atom‐level graphs, are first generated as the inputs. The residue‐level branch uses three hierarchical modules, i.e., the co‐evolution learning module, the hybrid CNN module, and the residue graph learning module, to learn residue‐level features. The other atom‐level branch employs the atom graph learning module to aggregate features of atoms in both backbone and sidechains, resulting in atom‐level representation. Next, the residue‐level and atom‐level features are fused through variational learning to refine the backbone information. Finally, the fused features are sent into the 3D Structure generator to predict the 3D all‐atom structure.

The whole framework of the proposed LightRoseTTA is illustrated in **Figure** **1**. Overall, it employs the backbone‐to‐all‐atom architecture for 3D structure prediction, which first predicts the backbone and then obtains the all‐atom structure by leveraging high backbone‐sidechain dependency. For one given amino acid sequence, three aspects of information, i.e., MSAs,^[^ ^20^, ^21^ ^]^ structure templates, and atom‐level graphs, are first generated as inputs of the LightRoseTTA. Next, a two‐branch network, consisting of the residue‐level and atom‐level branches, is constructed to model both inter‐residue interaction and sidechain influence for backbone conformation. The residue‐level branch first applies the co‐evolution learning module to alternately update MSA and template information in a co‐evolutional way. It outputs both residue features and paired inter‐residue relation. Then, a hybrid convolution neural network (CNN),^[^ ^22^ ^]^ performing convolutions by using both classic (unsymmetric) and symmetric convolutional kernels, is designed to learn pairwise inter‐residue representation. We further treat residue features and inter‐residue representation as nodes and edges, respectively, to construct a residue‐level graph. Subsequently, a graph transformer^[^ ^23^ ^]^ is employed to learn geometric representation by aggregating residue features according to inter‐residue edges. In contrast to RoseTTAFold,^[^ ^12^ ^]^ this residue‐level branch has two notable differences: 1) possessing a rather shallow architecture of much fewer co‐evolution blocks in the co‐evolution learning module; and 2) designing the symmetric convolutional kernels in the hybrid CNN to preserve the intrinsic symmetry of pairwise attributes (e.g., inter‐residue dihedrals ω: C_α_‐C_β_‐C_β_‐C_α_).

The other atom‐level branch additionally takes the sidechain effects into account and constructs the atom‐level graph by regarding atoms and bonds as nodes and edges. The atom‐level representation is then learned through a graph neural network (GNN)^[^ ^24^ ^]^ by aggregating features of atoms in both backbone and sidechains. The atom‐level branch is fused into the residue‐level branch through variational learning to inject sidechain awareness into backbone generation. Hence, the residue‐level representation can be further refined by adaptively considering the influence of atom‐level variation. After the two‐branch fusion, the resulting features are fed into the 3D‐Structure generator for both backbone and all‐atom structure prediction. Concretely, the coordinates of the backbone atoms (N, C_α_, C) are first predicted through the SE(3)‐Transformer.^[^ ^25^ ^]^ To further obtain the all‐atom structure, next, the sidechain atoms are bonded to the backbone and optimized based on the all‐atom potential energy.

For the model training, a constraint prior named backbone potential energy (BPE) is specifically proposed for backbone prediction. The BPE effectively regularizes the parameter optimization space, and enables a pretty shallow model to predict accurate backbone structures. Moreover, to make LightRoseTTA robust against homologous sequences, a new MSA dependency‐reducing strategy is further designed during the model training. In the training stage, the whole architecture can be optimized in an end‐to‐end manner through gradient backpropagation.

### 2.2 Predicting Protein Structures in CASP14 and CAMEO

**Figure 2:**
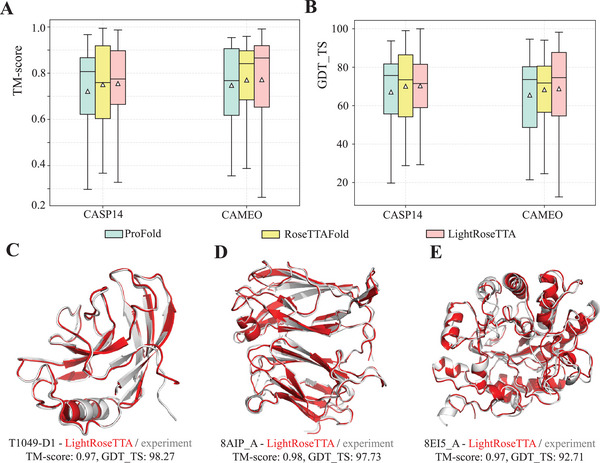
The performance comparison on the CASP14 and CAMEO datasets and the visualization examples of LightRoseTTA's prediction. A) The performance (TM‐score) on the CASP14 and CAMEO datasets. For each box in the figure, the center line, bottom line, and top line represent the median, first quartile, and third quartile, respectively. The horizontal lines along the top and bottom edges represent the maximum and minimum observations. Besides, the white triangle represents the average value. On the CASP14 dataset, the mean value of TM‐score for ProFold, RoseTTAFold, and LightRoseTTA are 0.722, 0.751, and 0.755, respectively. For CAMEO, the values of the three methods are 0.747, 0.77, and 0.772, respectively. B) The performance (GDT_TS) on the CASP14 and CAMEO datasets. For CASP14, the mean value of GDT_TS for ProFold, RoseTTAFold, and LightRoseTTA are 67.56, 70.07, and 70.34, respectively. For CAMEO, the values of the three methods are 66.09, 68.3, and 68.78, respectively. C) The LightRoseTTA's prediction of CASP14 domain T1049‐D1 (PDB code: 6Y4F) compared with the true (experimental) structure. D) The LightRoseTTA's prediction of CAMEO target 8AIP_A compared with the true (experimental) structure. E) The LightRoseTTA's prediction of CAMEO target 8EI5_A compared with the true (experimental) structure. “The prediction is colored red, and the true (experimental) structure is colored gray”.

To evaluate the performance of the LightRoseTTA for protein structure prediction, we first compare it with those methods named RoseTTAFold^[^ ^12^ ^]^ and ProFold^[^ ^26^ ^]^ on the popular CASP14^[^ ^15^ ^]^ and CAMEO^[^ ^16^ ^]^ datasets. For performance measurement, the widely adopted TM‐score^[^ ^17^ ^]^ and GDT_TS^[^ ^18^ ^]^ (the higher the better for both of them) are used as the metrics for protein structure prediction. The experimental results are shown in **Figure** **2**A and Figure 2B. Overall, our LightRoseTTA achieves competitive prediction performances on both CASP14 and CAMEO datasets. For the metric of TM‐score, it outperforms RoseTTAFold by obtaining 0.004 (0.755 of LightRoseTTA vs 0.751 of RoseTTAFold) performance gain on CASP14 and achieving 0.002 (0.772 of LightRoseTTA vs 0.77 of RoseTTAFold) higher performance on CAMEO. Meanwhile, LightRoseTTA outperforms ProFold on both CASP14 and CAMEO with performance improvements of 0.033 and 0.025, respectively. For the other metric of GDT_TS, similar with the comparison results of TM‐score, LightRoseTTA outperforms RoseTTAFold on CASP14 (70.34 of LightRoseTTA vs 70.07 of RoseTTAFold), and on CAMEO (68.78 of LightRoseTTA vs 68.3 of RoseTTAFold). Also, LightRoseTTA performs better than ProFold with the GDT_TS improvements of ≈2.78 and 2.69 on CASP14 and CAMEO, respectively. In order to more intuitively show the predictive performance of LightRoseTTA, we visualize several examples of LightRoseTTA‐predicted structures in Figure 2C–E, which intuitively show the high structural consistency between the predicted results (red) of our LightRoseTTA and the structure obtained through experiments (gray). Furthermore, we compare the prediction results of LightRoseTTA and RoseTTAFold with respect to the proportion of secondary structures (helices, beta‐fragments and loops), and the protein length of CAMEO proteins in Section S4 (Supporting Information), with the results shown in Figure S2 (Supporting Information).

### 2.3 Predicting Structures of Proteins with Insufficient Homologous Sequences

**Figure 3:**
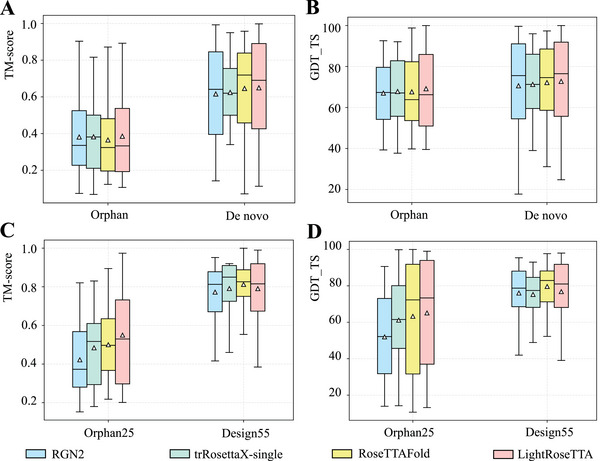
The performance comparison on the Orphan, De novo, Orphan25, and Design55 datasets. A) The performance (TM‐score) on the Orphan and De novo datasets. For each box in the figure, the center line, bottom line, and top line represent the median, first quartile, and third quartile, respectively. The horizontal lines along the top and bottom edges represent the maximum and minimum observations. Besides, the white triangle represents the average value. For the Orphan dataset, the mean values of TM‐score for RGN2, trRosettaX‐single, RoseTTAFold, and LightRoseTTA are 0.381, 0.382, 0.365, and 0.385, respectively. For the De novo dataset, the values of four methods are 0.615, 0.622, 0.642, and 0.649, respectively. B) The performance (GDT_TS) on the Orphan and De novo datasets. For the Orphan dataset, the mean value of GDT_TS for RGN2, trRosettaX‐single, RoseTTAFold, and LightRoseTTA are 67.04, 67.83, 67.64, and 69.15, respectively. For De novo dataset, the values of the four methods are 70.63, 71.65, 72.18, and 72.82, respectively. C) The performance (TM‐score) on the Orphan25 and Design55 datasets. For the Orphan25 dataset, the mean values of TM‐score for RGN2, trRosettaX‐single, RoseTTAFold, and LightRoseTTA are 0.422, 0.482, 0.491, and 0.549, respectively. For the Design55 dataset, the values of four methods are 0.772, 0.783, 0.812, and 0.787, respectively. D) The performance (GDT_TS) on the Orphan25 and Design55 datasets. For the Orphan25 dataset, the mean values of GDT_TS for RGN2, trRosettaX‐single, RoseTTAFold, and LightRoseTTA are 51.96, 61.09, 63.25, and 65.11, respectively. For the Design55 dataset, the values of four methods are 76.08, 75.24, 79.63, and 76.57, respectively.

To evaluate the robustness of our LightRoseTTA to proteins with insufficient homologous information, we specifically test the performance on four public datasets, including Orphan and De novo (human‐designed) in ref. [14] as well as Orphan25 and Design55 in ref. [13]. In these four datasets, protein samples generally possess rather limited or even no homologous sequences. To deal with these homolog‐insufficient proteins, two natural language model‐based methods named RGN2^[^ ^14^ ^]^ and trRosettaX‐single^[^ ^13^ ^]^ are previously proposed, respectively. Both of them are MSA‐free, which means that no homologous information is used. For a comprehensive comparison of the four datasets, we compare our results with a representative method named RoseTTAFold,^[^ ^12^ ^]^ as well as the two natural language model‐based methods RGN2 and trRosettaX‐single. For the Orphan and De novo (human‐designed) datasets in ref. [14], the comparison results are shown in **Figure** **3**A,B. [For samples in the four datasets, homologous sequences, although quite limited or none, are still searched from Uniref30^[^ ^4^ ^]^ released as of June 2020 and BFD.^[^ ^27^ ^]^ Therefore, the reported results of RoseTTAFold in Figure 3A–D are better than those in ref. [13, 14] with no homologous sequences used. In addition, we also show the result of predictive performance without using MSA in Figure S6 (Supporting Information).] Our LightRoseTTA outperforms all these comparison methods, i.e., RoseTTAFold, trRosettaX‐single and RGN2, on both Orphan and De novo datasets, with both higher values of TM‐score and GDT_TS. Moreover, the results on the recent Orphan25 and Design55 datasets^[^ ^13^ ^]^ are shown in Figure 3C,D. Accordingly, our LightRoseTTA achieves the best prediction performance on Orphan25 while gets lower performance only than RoseTTAFold on Design55. The comparison results verify the robustness of our LightRoseTTA to proteins with insufficient homologous information. To intuitively show the high structural consistency between the predicted and experimental structures, we visualize several examples of LightRoseTTA‐predicted structures in Figure S3 (Supporting Information). Moreover, we also show the prediction of some difficult targets in Figure S5 (Supporting Information).

### 2.4 Transferring LightRoseTTA to Predict Antibody on Rosetta Antibody Benchmark

The antibody is a special type of protein that plays an important role in human immunity. Different from the aforementioned proteins, the structural diversity of antibodies is primarily concentrated in those complementary determining regions (CDRs). Hence, directly using the model trained on general proteins may not obtain satisfactory performance on antibody data. To solve this problem, we transfer our LightRoseTTA to predict the antibody structure by further fine‐tuning the parameters based on antibody data, resulting in the model named LightRoseTTA‐Ab. We test the LightRoseTTA‐Ab on the Rosetta Antibody Benchmark dataset,^[^ ^19^ ^]^ and compare the performance with the results of DeepAb,^[^ ^19^ ^]^ IgFold,^[^ ^28^ ^]^ and Ablooper.^[^ ^29^ ^]^ For the model fine‐tuning, we use the same training data with DeepAb.^[^ ^19^ ^]^

**Figure 4:**
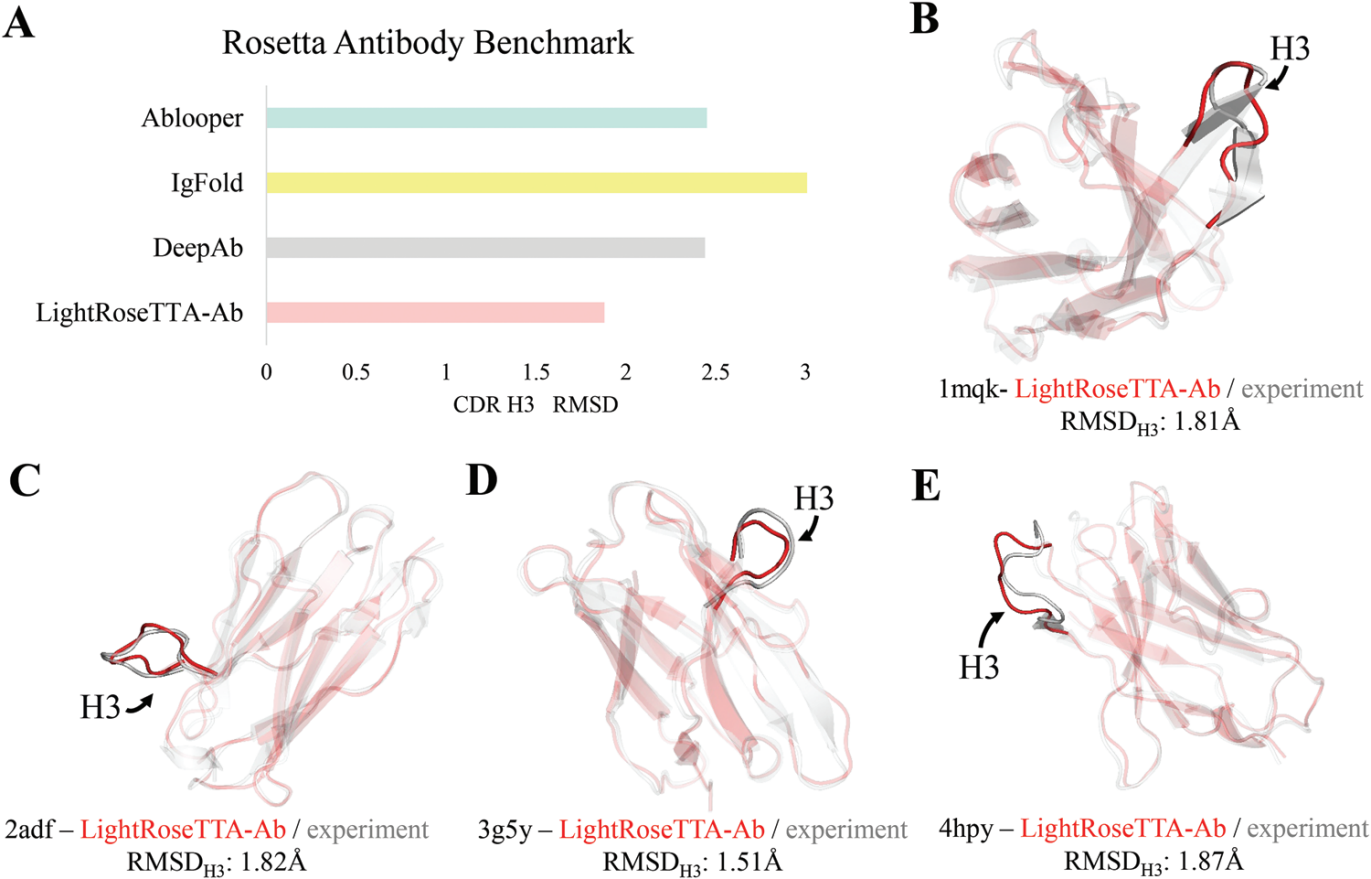
The performance comparison (RMSD‐Cα of CDR‐H3) on the Rosetta Antibody Benchmark and the visualization examples of the LightRoseTTA‐Ab's prediction. A) The performance (RMSD‐Cα of CDR‐H3) on the Rosetta Antibody Benchmark. B) The LightRoseTTA‐Ab's prediction of antibody 1mqk compared with the true (experimental) structure. C) The LightRoseTTA‐Ab's prediction of antibody 2adf compared with the true (experimental) structure. D) The LightRoseTTA‐Ab's prediction of antibody 3g5y compared with the true (experimental) structure. E) The LightRoseTTA‐Ab's prediction of antibody 4hpy compared with the true (experimental) structure. “The prediction is colored red, and the true (experimental) structure is colored gray”.

To measure the prediction performance, one specific metric for antibody named RMSD_H3_ is adopted. RMSD_H3_ measures the RMSD value (the lower the better) of the third complementary determining region (CDR) ring of the heavy chain (CDR‐H3). Specifically, the CDR‐H3 is a crucial region for antibodies and presents high structural diversity, which makes structural prediction rather difficult. **Figure** **4** shows the experimental results of different methods, as well as the visualized examples of predicted antibody structures. Our LightRoseTTA‐Ab obtains the RMSD_H3_ value of 1.9 Å, while the results of another two state‐of‐the‐art methods, i.e., Ablooper and DeepAb, are about 2.4 Å, and the RMSD_H3_ value of IgFold is ≈3.0 Å. The lowest RMSD_H3_ demonstrates the effectiveness of our LightRoseTTA‐Ab in promoting the structure prediction of antibodies. Moreover, we also visualize some prediction CDR‐H3 examples of antibodies in Figure 4B–E. As it is shown, the structural consistency of CDR‐H3 can be observed between the structure predictions (red) and experiments (gray), with the RMSD_H3_ values ≈1.5–1.9 Å. In addition, the ${\text{RMSD}}_{C_{\alpha }}$ results of all six CDRs are shown in Figure S13 (Supporting Information).

### 2.5 Ablation Study

**Figure 5:**
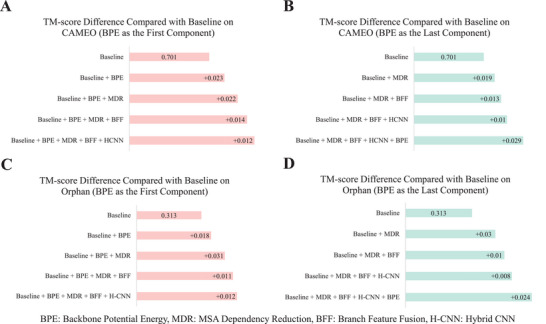
Ablation study on the CAMEO and Orphan dataset. A) TM‐score difference between the baseline and several ablation models on the CAMEO dataset. Each ablation model is constructed by adding one or several components to the baseline. Specifically, the BPE is the first component added. B) The TM‐score difference adds those components in a different order. Specifically, the BPE is the last‐added component. C) TM‐score difference between the baseline and several ablation models on the Orphan dataset. Specifically, the BPE is the first component added. D) TM‐score difference that adds those components in a different order, where the BPE is the last‐added component.

To evaluate how each component contributes to LightRoseTTA's performance, we conduct the ablation study on the CAMEO^[^ ^16^ ^]^ and Orphan datasets,^[^ ^14^ ^]^ and show the results in **Figure** **5**. The baseline takes a shallow co‐evolution learning module followed by a shallow SE(3)‐transformer. As observed in Figure 5A,B, on the CAMEO dataset, adding each component can effectively promote the prediction accuracy with the TM‐score improvements of 0.012−0.023 if the BPE is first imposed. Contrastively, without applying the BPE constraint first, less performance improvement is obtained by each same component, as shown in Figure 5B. The reason might be that the tremendous parameter searching space makes the model difficult to reach a suitable state. The parts of losses related to hard atom geometries (bond length, bond angle, etc) are difficult to decline if backbone potential energy (BPE) constraints are not used according to our experimental observation. Moreover, as shown in Figure 5C,D, for the Orphan dataset with insufficient homologous information, the BPE also effectively promotes the structure prediction with the TM‐score gain of ≈0.02. Specifically, on the Orphan dataset, we pay more attention to the MSA dependency reduction (MDR) strategy to evaluate its effectiveness. According to Figure 5C,D, the MDR improves the structure prediction performance of Orphan with the TM‐score gain of ≈0.03. This verifies the effectiveness of the MDR in improving the model's robustness to homologous sequences. Overall, in Figure 5, the ablation results verify the effectiveness of each component and the specific importance of MDR and BPE to boost the accuracy of protein structure prediction. The detailed description of each ablation component is described in the Experimental Section. Moreover, the experimental results of the other testing sets are shown in Figure S7 (Supporting Information).

## 3 Discussion

In this study, the light‐weight LightRoseTTA was proposed and comprehensively evaluated through both quantitative performance comparisons and visualization results. Generally, our LightRoseTTA can well handle the structure prediction for protein sequences with both sufficient and insufficient (even none) homologous sequences. Furthermore, our LightRoseTTA can be easily transferred to antibodies for structure prediction, and effectively promoted the prediction performance on CDR‐H3. Besides, considering the determinative relationship of protein structure for biological function, as our LightRoseTTA's prediction obtains high structural consistency with the true structure, we further discuss how they may contribute to the study of protein function in Section S5 (Supporting Information).

**Figure 6:**
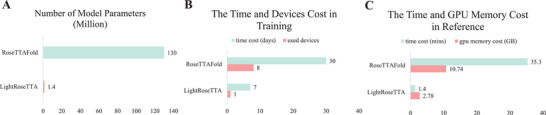
The parameter quantity, training cost, and testing cost of state‐of‐the‐art methods and LightRoseTTA. A) The number of model parameters. Our LightRoseTTA has the smaller model with 1.4M parameters, while the parameter number of RoseTTAFold is 130M. B) The time cost and devices used for training. LightRoseTTA can be trained with a single general GPU in one week, RosettaFold costs 30 days using 8 high‐speed V100 GPUs. C) The average time and GPU memory cost of testing one sequence on the CAMEO dataset. When predicting all‐atom structures, LightRoseTTA has an average prediction time of 1.4 minutes, and RoseTTAFold needs structural refinement with pyRosetta^[^ ^30^ ^]^ and costs a running time of 35.3 minutes. For the GPU memory requirement, LightRoseTTA has a smaller value at 2.78 GB, while RoseTTAFold requires 10.74 GB.

More importantly, our LightRoseTTA can be deployed to the general personal computer with a single GPU for efficient training. We further discuss the comparisons of the parameter quantity, time cost for training, as well as time and memory costs for prediction. The results are shown in **Figure** **6**A–C, where the representative high‐accuracy big model, i.e., RoseTTAFold, is compared. RoseTTAFold has a number of 130M parameters, and requires 30 days to train the framework using 8 high‐speed V100 GPUs. Our LightRoseTTA has only 1.4M parameters [We also test the performance of our model with different parameter quantities, and the results are shown in Figure S9 (Supporting Information).], and costs 7 days to train the model using only one NVIDIA 3090 GPU. Hence, with the high‐accurate performance guaranteed, our LightRoseTTA could largely shorten the research cycle for protein structure prediction under resource‐limited environment with general devices. In our further research, we will pay more attention to optimizing the inference code through GPU parallelism, further accelerating the prediction of protein sequences.

## 4 Experimental Section

### The Model Training

The LightRoseTTA was trained with the protein data released in the PDB before May 1, 2018, where 40340 non‐redundant protein domains are involved totally. More details of training and testing data are described in Section S3 (Supporting Information). Given the predicted structure from the 3D‐Structure Generator, to guide the model optimization, the network was primarily supervised by the five‐part losses, including 1) proposed BPE (bond length $Loss_{bb\_bl}$, bond angle $Loss_{bb\_ba}$ and dihedral $Loss_{bb\_bd}$) for the backbone generation; 2) the coordinate RMSD $Loss_{bb\_RMSD}$ of the predicted backbone structure; 3) mean squared error, i.e., $Loss_{bb\_pLDDT}$, of the predicted *C*_α_‐LDDT^[^ ^27^ ^]^ scores of the backbone; 4) inter‐residue distance and orientation losses (cross‐entropy), denoted as $Loss_{dist_{C_{\beta }}}$, *Loss*_ω_, *Loss*_θ_, and *Loss*_ϕ_, respectively; 5) the prior structural losses/constraints for all‐atom coordinates (bond length $Loss_{aa\_bl}$, bond angle $Loss_{aa\_ba}$ and dihedral $Loss_{aa\_bd}$). The details of loss functions are introduced in Section S2 (Supporting Information). Based on these losses, all model parameters could be optimized from scratch through backpropagation in an end‐to‐end manner. The training and testing could be run on a general PC with a single NVIDIA RTX 3090 (24GB GPU memory) card. More training details are described in Section S1 (Supporting Information), with the training and validation loss curve shown in Figure S10 (Supporting Information).

### Input Feature Generation

The input features of LightRoseTTA consisted of three parts, i.e., the MSA feature, the template information, and the atom‐level graph representation. To extract MSA features, for each protein sequence, the HHsuite tool^[^ ^31^ ^]^ was employed to retrieve homologous sequences against a protein sequence database named Uniref30^[^ ^4^ ^]^ (released before June 2020) and BFD.^[^ ^27^ ^]^ Based on these homologous sequences, MSA features were constructed as the token matrix (the tokens from 0 to 19 were represented as 20 types of amino acids, and the token 20 was the unknown amino acid) whose each column corresponded to the position in the alignment sequence, while each row indicated each sequence in the MSA. For the template construction, the 3D structures of homologous sequences were searched in the structure database named PDB100^[^ ^32^ ^]^ (released before March 2021, as same as RoseTTAFold). In the testing process, the templates corresponding to the query sequence itself were excluded.

The atom‐level graph treated atoms as nodes and between‐atom bonds as edges. Each node feature involved five aspects of information: 1) the type of amino acid (20 kinds of common amino acids); 2) the type of heavy atom (C, N, O, S); 3) the index of heavy atom in amino acid; 4) the label to determine whether each atom belongs to the backbone or side chain; and 5) initial 3D‐coordinates of the atom in the unfolded state. Among them, the first four items were represented as 20D, 4D, 14D, and 2D one‐hot vectors, respectively. Finally, all these parts of features were concatenated as a 43D vector.

### The Residue‐Level Branch

The residue‐level branch of this model took the Multiple Sequence Alignment (MSA) and template as inputs and comprised three key learning modules: co‐evolution learning, hybrid CNN, and residue graph learning. In the co‐evolution learning module, the MSA was tokenized and embedded as a feature vector, while pair features were generated by extracting pairwise distances and orientations from the template. To capture the co‐evolutionary information between the MSA and pairs, an interactive update mechanism was employed. For the update from the MSA to the pair, the outer product of the MSA was calculated and added to the pair, which effectively updated the pair based on the coevolution signal from the MSA. Conversely, for the update from pair to MSA, the cross‐attention between the MSA and pair was calculated, which transferred the geometric information of the pair to the MSA. Additionally, self‐attention was computed for both the MSA and the pair to facilitate self‐updating. This process yielded both residue features and pairwise inter‐residue relations. In addition, the architecture of the update mechanism is shown in Figure S4A–D (Supporting Information).

After the co‐evolution stage, a novel hybrid Convolutional Neural Network (CNN) that combines both symmetric and unsymmetric convolutional kernels was introduced. This design allowed for the learning of inter‐residue geometries, encompassing symmetric components such as inter‐residue distance ($d_{C_{\beta }}$: C_β_‐C_β_) and inter‐residue dihedral (ω: C_α_‐C_β_‐C_β_‐C_α_), as well as unsymmetric components like inter‐residue dihedral (θ: N‐C_α_‐C_β_‐C_β_) and inter‐residue planar angle (ϕ: C_α_‐C_β_‐C_β_). Moreover, the architecture of hybrid CNN is shown as in Figure S4E (Supporting Information).

Subsequently, based on the residue features and their inter‐residue representations, a residue‐level graph was constructed, with the features serving as nodes and the inter‐residue relations as edges. Finally, a graph transformer^[^ ^23^ ^]^ was employed for geometric representation learning. This involved learning multi‐head attention coefficients through a message‐passing procedure, enabling effective node and edge aggregation. The initial backbone structure was generated by mapping each node feature to the Cartesian coordinates of three atoms: N, C_α_, and C.

The architecture of the designed hybrid CNN, where two types of convolutional kernels, including both classic (unsymmetric) and symmetric ones, were used (shown in Figure S1A, Supporting Information). Specifically, the symmetric kernels could be constructed through the summation of the unsymmetric kernels and their transposes. For the convolutional learning, the unsymmetric kernels were used for the inter‐residue dihedral θ and inter‐residue planar angle ϕ, while the symmetric kernels count for the inter‐residue distance $d_{C_{\beta }}$ and inter‐residue dihedral ω. By virtue of the symmetric convolution, the symmetries of those inter‐residue geometries (i.e., $d_{C_{\beta }}$: C_β_‐C_β_ and ω: C_α_‐C_β_‐C_β_‐C_α_) could be well preserved. In detail, the symmetric convolution contained three blocks, and each block had a 3 × 3 symmetric convolution kernel layer, with a BatchNorm layer and an activation exponential linear unit (ELU). Formally, the symmetric convolution can be written as:

(1) $$\tilde{W}=\frac{1}{2}\left(W+W^{⊤}\right),$$

(2) $$A_{R}^{\left(l\right)}=\sigma \;\left(conv2d\left(A_{R}^{\left(l-1\right)},\tilde{W},b\right)\right),$$

where **W** is the learnable convolution kernel with **W** ^⊤^ denoting the corresponding transpose, **b** is the bias, and σ is the ELU activation. **A**_*R*_ denotes the matrix of inter‐residual relationship, and *l* is the layer number.

### The MSA‐Dependency Reduction Strategy

The MSA Dependency Reduction (MDR) strategy was proposed to enhance the robustness in handling insufficient Multiple Sequence Alignment (MSA) data. The experimental results with/without using MSA are shown in Figure S11 (Supporting Information), with other methods such as RoseTTAFold compared. **Algorithm** **1** outlines the key steps of this strategy. Two MSA selection approaches, namely, top‐k selection and two‐stage sampling, were utilized based on the probability of a binomial distribution (probability hyper‐parameter). For example, if a 0.3‐probability binomial distribution was adopted, the top‐k selection was applied to 30% of the training data, while the two‐stage sampling method was used for the remaining training data.

In the top‐k selection approach, the top 101 sequences were chosen (including one query sequence and 100 homologous sequences) from the “.a3m” file, which represents the MSA results obtained through HHsuite search. For the two‐stage sampling the number of selected homologous sequences were first determined, denoted as k. To do this, a random number, k, was generated from a uniform distribution in the range of 1–min(nH, 100), where nH represents the total number of searched homologous sequences. Subsequently, k homologous sequences were randomly selected as the input for MSA. This two‐stage sampling approach allowed for the inclusion of a limited number of homologous sequences during the training process, thereby enhancing the model's robustness against situations of insufficient homologous sequences. Notably, when k equals 1, only the protein sequence itself was utilized as the MSA input, which corresponded to the scenario of predicting orphan proteins. In addition, the performances of Gaussian sampling were compared, stratified sampling and uniform sampling strategies for MDR. The results are shown in Figure S8 (Supporting Information).

Algorithm 1 **MSA Dependency Reduction.**

**Table:** 

**Input**: MSA token matrix;	
**Output**: Augmented MSA token matrix;	
1:	sample_flag←random_number;
2:	**if** sample_flag<0.3 **then**
3:	n←len(MSA_token_matrix);
4:	*k* ← *Uniform*[1, *min*(*n*_*H*_, 100)];
5:	MSA_index←random_sample(n,k);
6:	augmented_MSA←MSA_token_matrix[MSA_index]
7:	**end if**
8:	**if** sample_flag>=0.3 **then**
9:	augmented_MSA←MSA_token_matrix[:101]
10:	**end if**

### The Atom‐Level Branch

Considering that different sidechains might pose various influences on backbone conformation, the atom‐level branch was additionally constructed to make full use of the sidechain information. In this branch, atom‐level graphs were first constructed for the common 20 kinds of amino acids by regarding atoms as nodes and bonds as edges. Each amino acid was represented by a graph structure, with atoms serving as nodes and chemical bonds as edges. These individual amino acid atom graphs were then integrated into a larger protein atom graph by connecting them according to the amino acid sequence. In this process, the principle of dehydration and condensation need to be considered. Specifically, a molecule of water (nodes corresponding to two certain hydrogen atoms and one oxygen atom) should be removed when one amino acid molecule binds with another adjacent amino acid molecule. The peptide bond “–CO–NH–” connected two adjacent amino acid molecules. Then, a three‐layer GNN block^[^ ^24^ ^]^ was used to learn the representation of atoms so that the sidechain information could be propagated to the backbone atoms. Each GNN layer was constructed as *X* ^(*l* + 1)^ = *GNN*(*A*, *X* ^(*l*)^), where *X* ^(*l*)^ represents the feature matrix at the *l*‐th layer, and *A* is the adjacent matrix. To enhance the representation capacity, shortcut connections were introduced among these GNN layers. This approach enabled the effective integration of sidechain information, allowing it to influence the representation and prediction of backbone conformation.

### The Two‐Branch Fusion

A variational learning process was designed to learn weighting factors to fuse the residue‐level and atom‐level branches (the details are shown in Figure S1B, Supporting Information). Hereby, the residue features could be refined by mining local atom‐level structures that affect inter‐residue bonding, e.g., torsion and folding. Here, $V^{r}={\left[v_{1}^{r},\text{…},v_{L}^{r}\right]}^{⊤}\in R^{L\times d}$ (*L* is the number of residues) was used to denote the residue feature matrix, and $V^{a}={\left[v_{1}^{a},\text{…},v_{N}^{a}\right]}^{⊤}\in R^{N\times d}$ (*N* is the number of atoms) to denote the atom feature matrix. In the fusion process, the atom‐residue similarity matrix $S\in R^{L\times N}$ was first calculated, where the element in the *i*‐th row and *j*‐th column is formulated as $s_{ij}=\frac{{\left(v_{i}^{r}\right)}^{⊤}v_{j}^{a}}{∥v_{i}^{r}∥\;∥v_{j}^{a}∥}$. *s*_*ij*_ represents the relationship between the *i*‐th residue and *j*‐th atom in the protein. The fusion process is described in **Algorithm** **2**.

Algorithm 2 **Branch Feature Fusion.**

**Table:** 

**Input**: residue graph node feature $G^{r}\in R^{M\times d}$, atom graph node feature $G^{a}\in R^{N\times d}$;	
**Output**: residue graph fused feature **G^r^**_**fused**_;	
1:	$similarity\_matrix\leftarrow cosine\_similarity(G^{r},G^{a})$;
2:	sampling_filter←Bernoulli_sampling(similarity_matrix);
3:	${similarity\_matrix}^{\prime }\leftarrow sampling\_filter\cdot similarity\_matrix$;
4:	$G_{updated}^{a}\leftarrow {similarity\_matrix}^{\prime }\times G^{a}$;
5:	$G_{fused}^{r}\leftarrow F\left(\left[G^{r}∥G_{updated}^{a}\right]\right)$;

Considering the complicated influence of atoms on the backbone conformation, we resort to probabilistic reasoning to select salient atoms that may influence structural bonding, so as to refine residue features. Given the atom‐residue relationship **S**, a set of random variables were derived, denoted as Z, by learning the posterior probability $p(Z|V^{r},V^{a},S)$. Each element $z_{ij}\in Z$ followed the Bernoulli distribution, denoted as *z*_*ij*_ ∼ *B*(*p*_*ij*_), where *p*_*ij*_ represents the probability that the *j*‐th atom may influence the structural bonding of the *i*‐th residue. However, the posterior probability $p(Z|V^{r},V^{a},S)$ was usually intractable. For this problem, inspired by the reparameterization trick in ref. [33], the Bernoulli reparameterization was developed to derive the posterior probability $p(Z|V^{r},V^{a},S)$. Based on Z, salient atom features were selected for each residue. These selected atom features were concatenated with the corresponding residue feature and then fed into the 3D‐Structure generator for prediction. Furthermore, the architecture of the fusion process is shown in Figure S4G (Supporting Information).

### Backbone Potential Energy

In order to better train the LightRoseTTA, a constraint named backbone potential energy (BPE) was proposed. It regularized the structure of the protein backbone through the constraints of bond lengths, bond angles, and dihedrals. Specifically, the BPE focused on three bond lengths (N–*C*_α_, N–C, *C*_α_–C), three bond angles (N–*C*_α_–C, *C*_α_–C–N, C–N–*C*_α_), and three dihedrals (N–*C*_α_–C–N, *C*_α_–C–N–*C*_α_, C–N–*C*_α_–C). More details are provided in Section S2 (Supporting Information).

### The 3D‐Structure Generator

The 3D‐Structure generator transformed the residue features to all‐atom coordinates through a two‐stage inference. In the first stage, the multi‐layer perceptron was used to project residue features into the rough 3D coordinates, i.e., the coordinates of backbone N, *C*_α_, and C atoms for each residue. Then, a shallow SE(3)‐transformer was employed to further refine the backbone atoms. The architecture of refinement is shown in Figure S4F (Supporting Information). In the second stage, the sidechain atoms were first bonded to the backbone. Then, an all‐atom structural optimization mechanism, motivated by molecular dynamics, was proposed to guide the learning of all‐atom structure. Concretely, the proposed all‐atom optimization mechanism consisted of two parts, i.e., the prior structural constraints of side chains and the Langevin integrator.^[^ ^34^ ^]^ The prior structural constraints of side chains regularize the bond length, bond angle and dihedral, and are described in Equation (S3) (Supporting Information). Moreover, through a mild Brownian motion, the Langevin integrator prevented atoms from excessively approaching each other after the folding process.

## Conflict of Interest

The authors declare no conflict of interest.

## Supporting information

Supporting Information

## Data Availability

The data that support the findings of this study are openly available in LightRoseTTA_test_data at https://zenodo.org/records/10189502, reference number 10189502.
