# Supplementary material for: LightRoseTTA: High‐Efficient and Accurate Protein Structure Prediction Using a Light‐Weight Deep Graph Model
Source: Adv Sci (Weinh). 2025 Mar 25;12(19):2309051. doi: 10.1002/advs.202309051 (PMC12097069; doi:10.1002/advs.202309051)
Supplement: Supplementary file 1 — Supporting Information [file ADVS-12-2309051-s001.pdf]

ADVANCED SCIENCE
Open Access

Supporting Information

for *Adv. Sci.*, DOI 10.1002/advs.202309051

LightRoseTTA: High-Efficient and Accurate Protein Structure Prediction Using a Light-Weight Deep Graph Model

Xudong Wang, Tong Zhang, Guangbu Liu, Zhen Cui, Zhiyong Zeng, Cheng Long, Wenming Zheng* and Jian Yang*

Supporting Information of "LightRoseTTA: High-efficient and Accurate Protein Structure Prediction Using a Light-weight Deep Graph Model"

Xudong Wang† *Tong Zhang*† *Guangbu Liu* *Zhen Cui** *Zhiyong Zeng* *Cheng Long* *Wenming Zheng** *Jian Yang*

X. Wang, T. Zhang, G. Liu, Z. Cui, J. Yang
School of Computer Science and Engineering
Nanjing University of Science and Technology
Nanjing 210094, China
Email Address: zhen.cui@njust.edu.cn
Z. Zeng
School of Automation
Nanjing University of Science and Technology
Nanjing 210094, China
C. Long
School of Computer Engineering
Nanyang Technological University
No. 50, Nanyang Avenue 639798, Singapore
W. Zheng
School of Biological Science & Medical Engineering
Southeast University
Nanjing 210096, China
Email Address: wenming_zheng@seu.edu.cn

1 Training details

The proposed LightRoseTTA is trained using a single NVIDIA RTX 3090 (24GB GPU memory) card within only one week. It has an light-weight model with about only 1.4M parameters. The following hyper-parameters are used:

- The size of MSA, pair, template features in hidden layers for co-evolution learning: 32
- The number of self-attention heads of MSA, pair, and template for co-evolution learning: 2
- The number of attention heads for MSA updates based on pair for co-evolution learning: 4
- The number of attention heads for pair updates based on MSA for co-evolution learning: 2
- The number of blocks for co-evolution learning: 6
- The size of symmetric kernel for hybrid CNN: 3×3
- The number of symmetric CNN blocks for hybrid CNN: 3
- The size of node and edge features in hidden layers of graph transformer for residue-level graph learning: 64
- The number of graph transformer blocks for residue-level graph learning: 3
- The number of attention heads on graph transformer for residue graph learning: 4

†Xudong Wang and Tong Zhang contribute equally to this work.
*Corresponding Author

- The size of node features for atom-level graph learning: 64
- The number of GNN blocks for atom graph learning: 3
- The size of input node and edge features for SE(3)-Transformer: 16
- The configuration of SE(3)-Transformer: 2 layers with 8 channels, 1 attention head, and up to representation degree 2
- Learning rate: 0.0005 with 50% learning rate decay if the loss does not decrease over 3 epochs
- Batch size: 4 in total (single GPU for training, 4 gradient accumulation steps)
- Weight decay: 0.0005

2 Loss functions

The training process consists of two stages, i.e. (1) the backbone training stage, and (2) the all-atom training stage. The backbone training is first conducted to provide a good initial structure. Hence, those sidechains can be further well-bonded for full-atom structure prediction. The backbone loss function, i.e. $Loss_{bb}$, can be formulized as:

$$Loss_{bb} = Loss_{BPE} + 0.3Loss_{dist_{C_\beta}} + 0.5(Loss_\omega + Loss_\theta + Loss_\phi + Loss_{bb\_RMSD}) + 0.01Loss_{bb\_pLDDT}, \tag{1}$$

where

$$Loss_{BPE} = 0.005(Loss_{bb\_bl} + Loss_{bb\_ba} + Loss_{bb\_bd}). \tag{2}$$

$Loss_{bb\_bl}$ is the RMSD loss between bond length (N-C_α, N-C, C_α-C) computed from predicted coordinates of backbone structure and true bond length. For $Loss_{bb\_ba}$ and $Loss_{bb\_bd}$, we compute the RMSD loss of bond angles (N-C_α-C, C_α-C-N, C-N-C_α) and dihedrals (N-C_α-C-N, C_α-C-N- C_α, C-N-C_α-C), respectively.

$Loss_{bb\_RMSD}$ is the coordinate RMSD loss between predicted and true structure. $Loss_{bb\_pLDDT}$ is the RMSD loss of lDDT score of C_α. $Loss_{dist_{C_\beta}}, Loss_\omega, Loss_\theta$ and $Loss_\phi$ are the cross-entropy loss for inter-residue distance and orientations. In particular, as performed by RoseTTAFold [1], we divide the inter-residue C_β distance range into 36 intervals, i.e., (2.5 Å, 3.0 Å), (3.0 Å, 3.5 Å), $\cdots$, and (20.0 Å, 20.5 Å). For ω and θ in orientation, we also divide it into 36 bins from 0 to 360 degrees. But for planar angle ϕ, because it ranges from 0 to 180 degrees, it's divided into 18 parts. After mapping the distance and orientation to the intervals, the values would be rounded off to the adjacent integers. So for distance and two dihedrals, the final number of classes is 37, and for the planar angle is 19.

In the all-atom training stage, sidechains are bonded to the backbone. Specifically, we set all these sidechain atom coordinates as optimizable parameters, and optimize them based on the following all-atom loss function:

$$Loss_{aa} = Loss_{bb} + 0.01(Loss_{aa\_bl} + Loss_{aa\_ba} + Loss_{aa\_bd}). \tag{3}$$

$Loss_{aa\_bl}, Loss_{aa\_ba}$ and $Loss_{aa\_bd}$ are the potential energy constrains (bond length, bond angle and dihedral) on sidechain and are also based on RMSD loss.

3 Benchmark datasets.

In this study, the training samples come from the training data used by ProFold [2] and trRosetta [3]. The ProFold uses the total number of 31247 domains, while trRosetta uses 9093 domains after removing the

duplicate data with ProFold. All the domains of proteins are divided according to the CATH database (as of May 1. 2018). Finally, the training dataset of LightRoseTTA contains 40340 non-redundant domains.

We test our proposed model on seven datasets: CASP14, CAMEO, Orphan, De novo, Orphan25, Design55, and Rosetta Antibody Benchmark. The used samples of CASP14 dataset are composed of the available domain data and the target data could be split correctly by domain defination index in the download area of CASP (the competition of Critical Assessment of Techniques for Protein Structure Prediction) website, where 32 available domains are finally got. The CAMEO test set consists of 130 structures used in the ongoing CAMEO assessment (between August 2023 to September 2023). For proteins with limited homologous sequences, we use four datasets from two studies: Orphan and De novo from [4], Orphan25 and Design55 from [5]. Orphan consists of 77 proteins having no homologs across Uniref30, PDB70, and MGnify simultaneously, and De novo has 149 de novo designed synthetic proteins using computed parameterized energy functions. Orphan25 contains 25 proteins (released from PDB after May 2020) which are searched against the sequence database UniRef50_2018_03 and no homologous sequence is returned, and Design55 consists of 55 de novo or computer designed proteins. For antibody data, we use the same testing samples as those of DeepAb [6] in the Rosetta Antibody Benchmark. There are 47 antibodies in total (each antibody contains both heavy chain and light chain).

4 The performance analysis with respect to different structure patterns on CAMEO

In order to study the performance with respect to different structure patterns, we use the DSSP method [7] to determine each secondary structure element (helices, beta-fragments and loops) in the PDB structure of the CAMEO dataset. As shown in Figure S2A-B, we can observe that our LightRoseTTA obtains better structure prediction performance than RoseTTAFold on proteins rich in helices, while RoseTTAFold performs better on beta-fragment-rich proteins. On loops, there is no clear winner between RoseTTAFold and LightRoseTTA. We also analyze the performance of proteins with different lengths in Figure S2C-D. In each length bin, there exist proteins for which LightRoseTTA's prediction results are better than those of RoseTTAFold. Overall, for the proteins with lengths up to 300, our LightRoseTTA possesses the performance advantage compared with RoseTTAFold.

5 Providing insights into biological function.

Experimental determination of protein structure can provide significant insights for understanding biological function and mechanism. As the predictions of LightRoseTTA reach better structural consistency with the true structure, we discuss whether they may also contribute to the study of protein function.

Bacterial infection is one of the causes of human disease. Specifically, for the infectivity of Plasmodium mirabilis, Mannose-resistant Proteus-like pili (MR/P) plays a vital role. T1049-D1 (PDB code: 6Y4F), shown in Figure 2C, is the tip adhesin of MR/P and its transition metal center is essential for MR/P fiber-mediated biofilm formation. Structurally, the transition metal center with Zn^{2+} is coordinated by three histidine residues (His-72, His-74, His-117) and a ligand. Accordingly, the LightRoseTTA-predicted structure of the metal center has the RMSD value of only 1.36 Å against the true structure. Hence, the accurate structure prediction of LightRoseTTA may facilitate the understanding of MR/P fiber-mediated biofilm formation mechanism, and further provide suggestions for preventing the infection of Plasmodium mirabilis.

An enzyme is a protein or RNA produced by living cells that are highly substrate-specific and catalytic. The catalysis of enzymes depends on the integrity of the primary and spatial structure of enzyme molecules. 5BVL_A (shown in Figure S10D) is a de novo designed Triose-phosphate isomerase (commonly abbreviated as TPI or TIM) that catalyzes the conversion of triose-phosphate isomers between dihydroxyacetone phosphate and D-type glyceraldehyde-3-phosphate. It plays an important role in glycolysis and is essential for efficient energy generation. LightRoseTTA's prediction of 5BVL_A presents a beta-barrel

fold with the active site of the enzyme located in the center of the "barrel". Specifically, the repeating α- and β- structures of the TIM-barrel construct a scaffold providing active sites for catalysis.

Antibodies are proteins secreted primarily by plasma cells and used by the immune system to identify and neutralize pathogens. 2adf, shown in Figure 4C, is the antithrombotic monoclonal antibody directed against the von Willebrand factor A3-domain. It effectively inhibits the interaction between von Willebrand factor and collagens, which is a prerequisite for blood platelet to adhere to the injured vessel walls at high-shear sites. The paratope predominantly consists of two short sequences in the heavy chain CDR1 (Asn-31 and Tyr-32) and CDR3 (Asp-99, Pro-101, Tyr-102, and Tyr-103). According to the heavy chain structure predicted by LightRoseTTA-Ab (shown in Figure 4C), these two short sequences form one patch on the surface of the antibody. Meanwhile, Trp-50 of the heavy and His-49 of the light chain are both situated adjacent to the patch, playing ancillary roles in antigen binding. This may provide some useful cues to the development of the novel antithrombotic peptidoid drug.

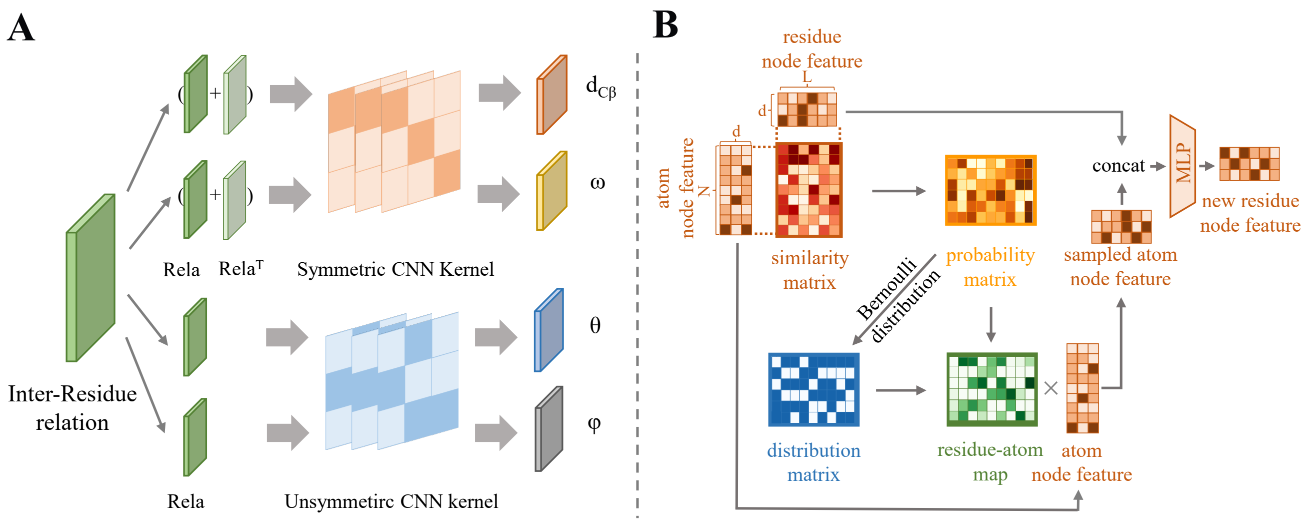

Figure S1: **The architecture of hybrid CNN and feature fusion mechanism. A)** The architecture of the hybrid CNN. d_{C_β} (C_β-C_β distance) and dihedral ω (C_α-C_β-C_β-C_α) are symmetric geometries. They are learnt with symmetric convolutional kernels. The dihedral θ (N-C_α-C_β-C_β) and planar angle ϕ (C_α-C_β-C_β) are unsymmetric geometries. They are learnt with unsymmetric convolutional kernels. **B)** The architecture of the feature fusion. In this module, the cosine similarity matrix (L*N) between the residue (L*d) feature and atom (N*d) feature is first calculated, and further activated with a softmax function to learn the probability matrix. Then, the Bernoulli distribution is derived based on the probability matrix, and fused with the probability matrix through the element-wise multiplication. This process results in a salient residue-atom map. Finally, the atom feature is multiplied with the salient residue-atom map, and concatenated with the corresponding residue node feature.

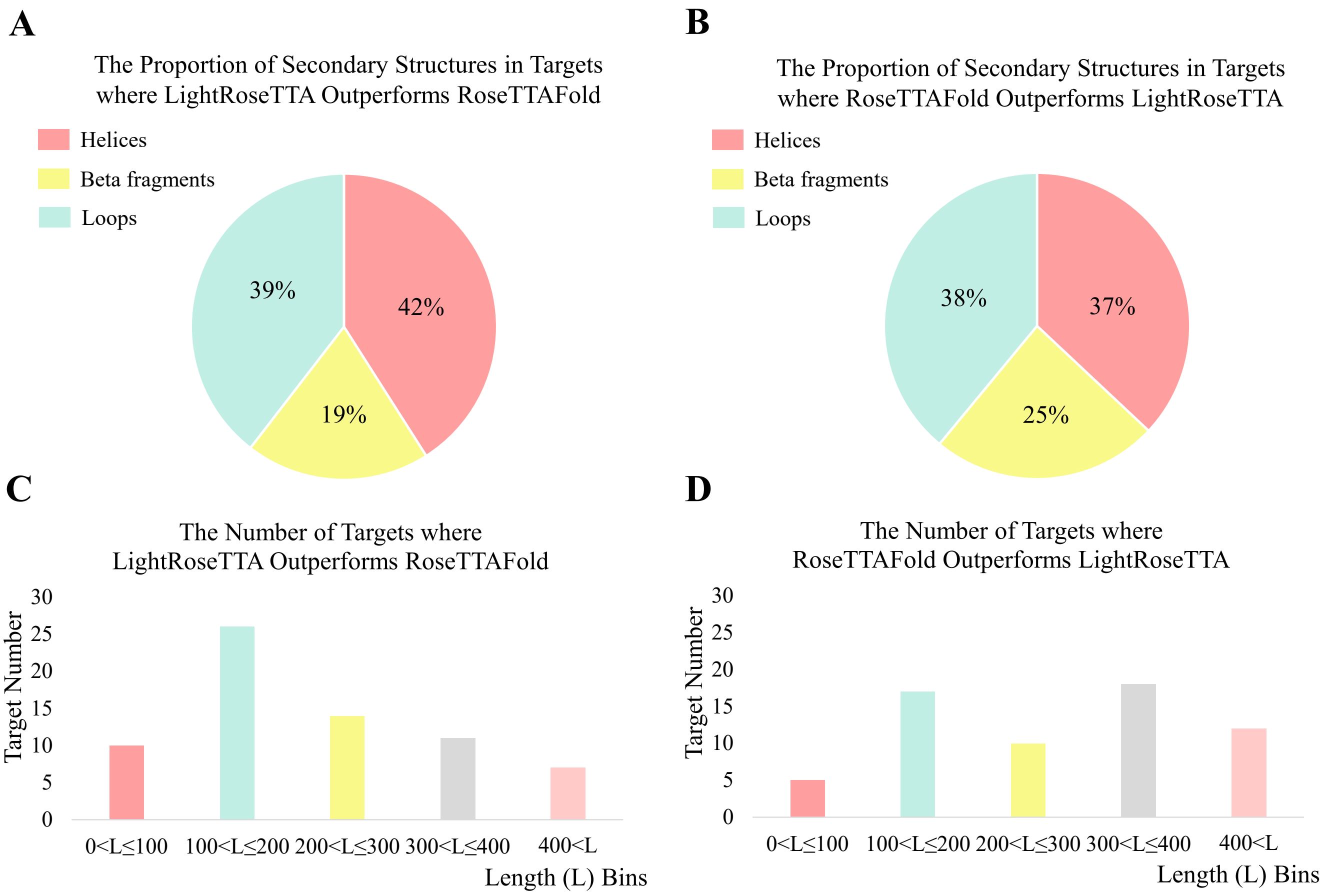

Figure S2: **The performance analysis with respect to different structure patterns on CAMEO. A)** The proportion of helices, beta fragments, and loops of proteins where LightRoseTTA predicts better. **B)** The proportion of helices, beta fragments, and loops of proteins where RoseTTAFold predicts better. **C)** The length distribution of proteins where LightRoseTTA predicts better. **D)** The length distribution of proteins where RoseTTAFold predicts better.

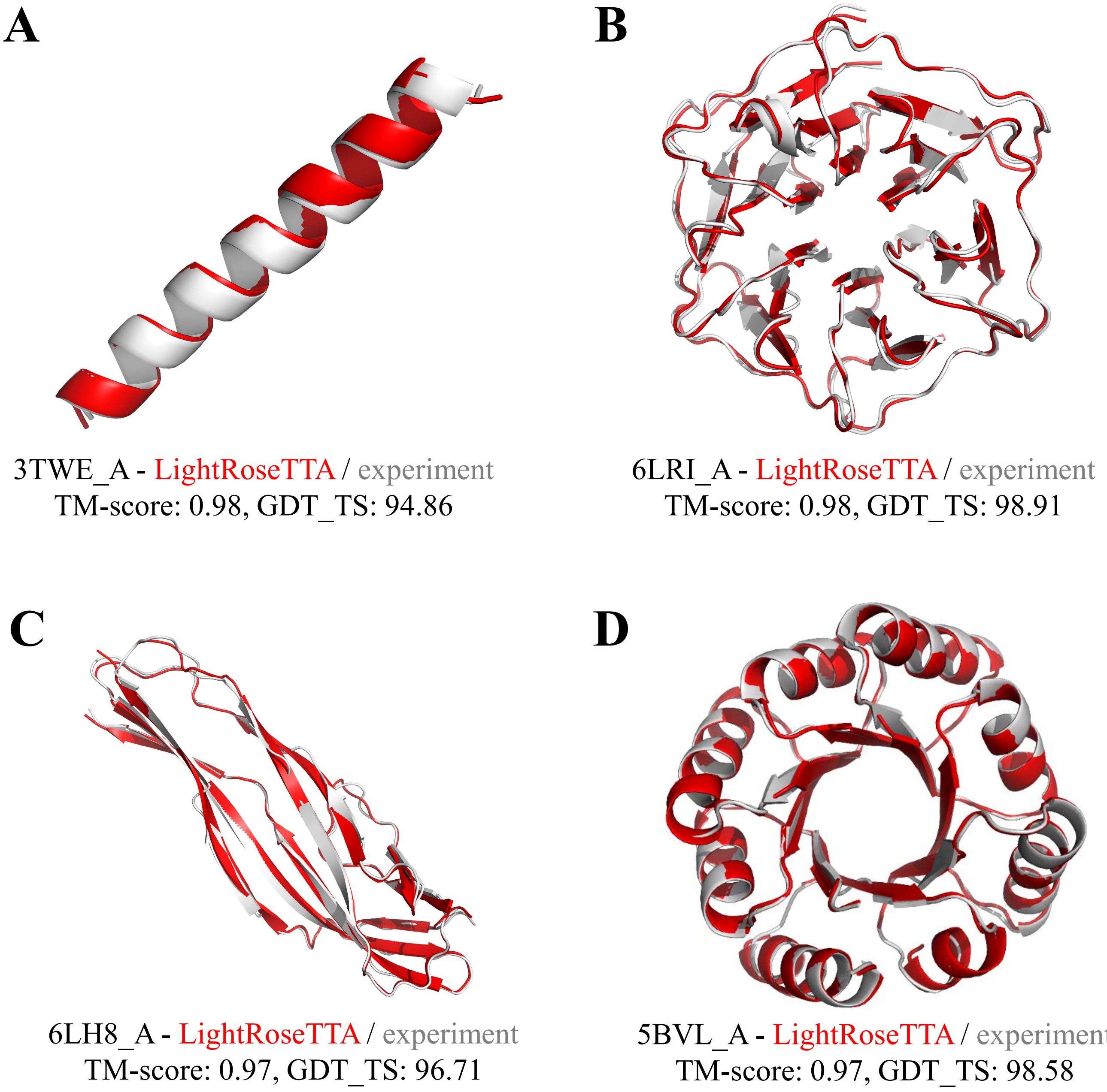

Figure S3: **The visualization of LightRoseTTA's predicted protein structures with TMscore and GDT on proteins with insufficient homologous sequences. A)** The LightRoseTTA's prediction of Orphan target 3TWE_A compared with the true (experimental) structure. The TMscore is 0.98 and GDT_TS is 94.86. **B)** The LightRoseTTA's prediction of Denovo target 6LRI_A compared with the true (experimental) structure. The TMscore is 0.98 and GDT_TS is 98.91. **C)** The LightRoseTTA's prediction of Orphan25 target 6LH8_A compared with the true (experimental) structure. The TMscore is 0.97 and GDT_TS is 96.71. **D)** The LightRoseTTA's prediction of Design55 target 5BVL_A compared with the true (experimental) structure. The TMscore is 0.97 and GDT_TS is 98.58. *The prediction is colored red, and the true (experimental) structure is colored gray.*

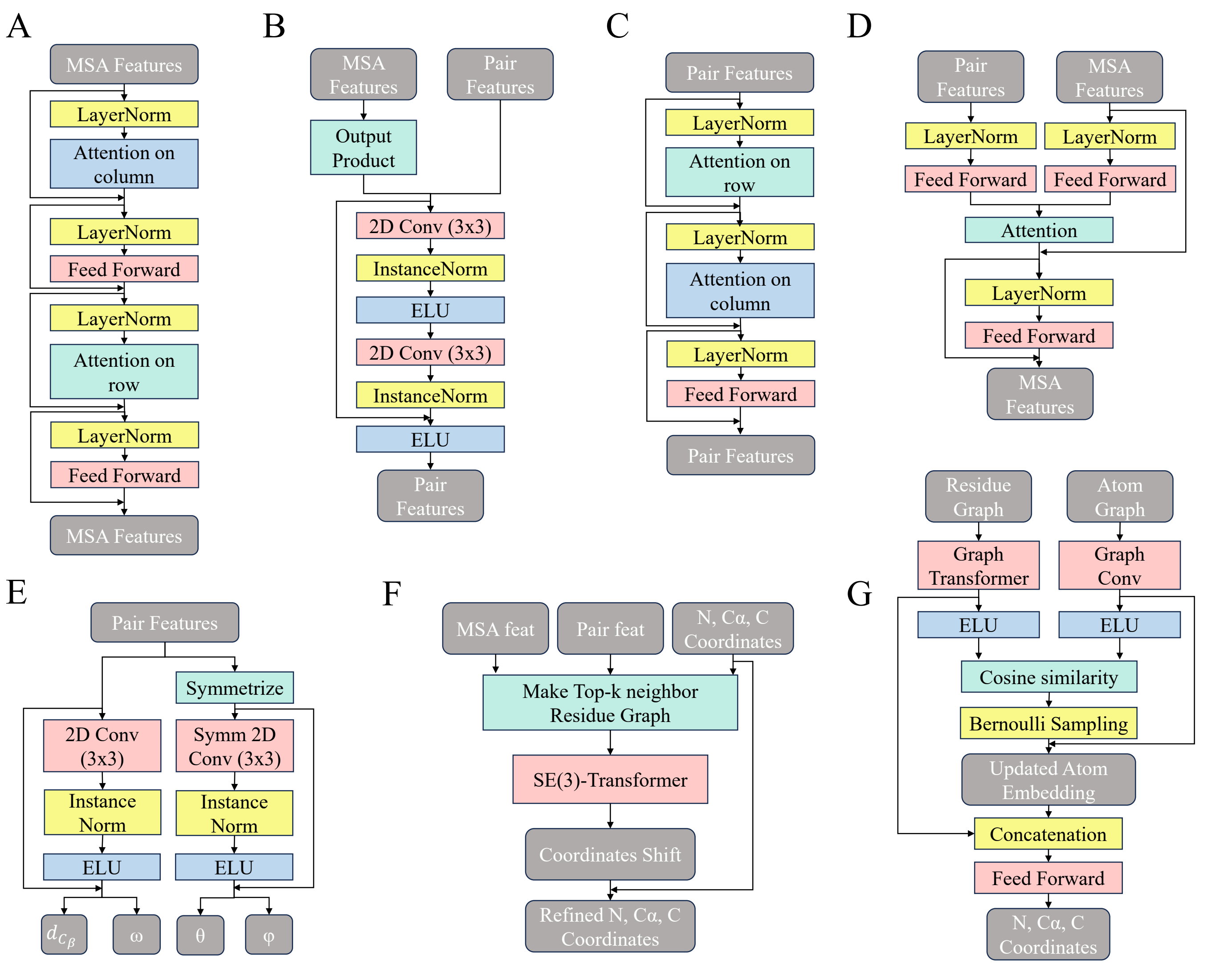

Figure S4: **The architecture of network modules of LightRoseTTA. A)** The architecture of the self-update of MSA via self-attention. **B)** The architecture of updating the pair data through MSA outer-products. **C)** The architecture of the self-update of pair feature via axial-attention. **D)** The architecture of updating the MSA feature based on attention derived from pair data. **E)** The architecture of hybrid convolution neural network. **F)** The architecture of protein structure refinement based on SE(3)-transformer. **G)** The architecture of the residue graph neural network, atom graph neural network and two-branch fusion mechanism.

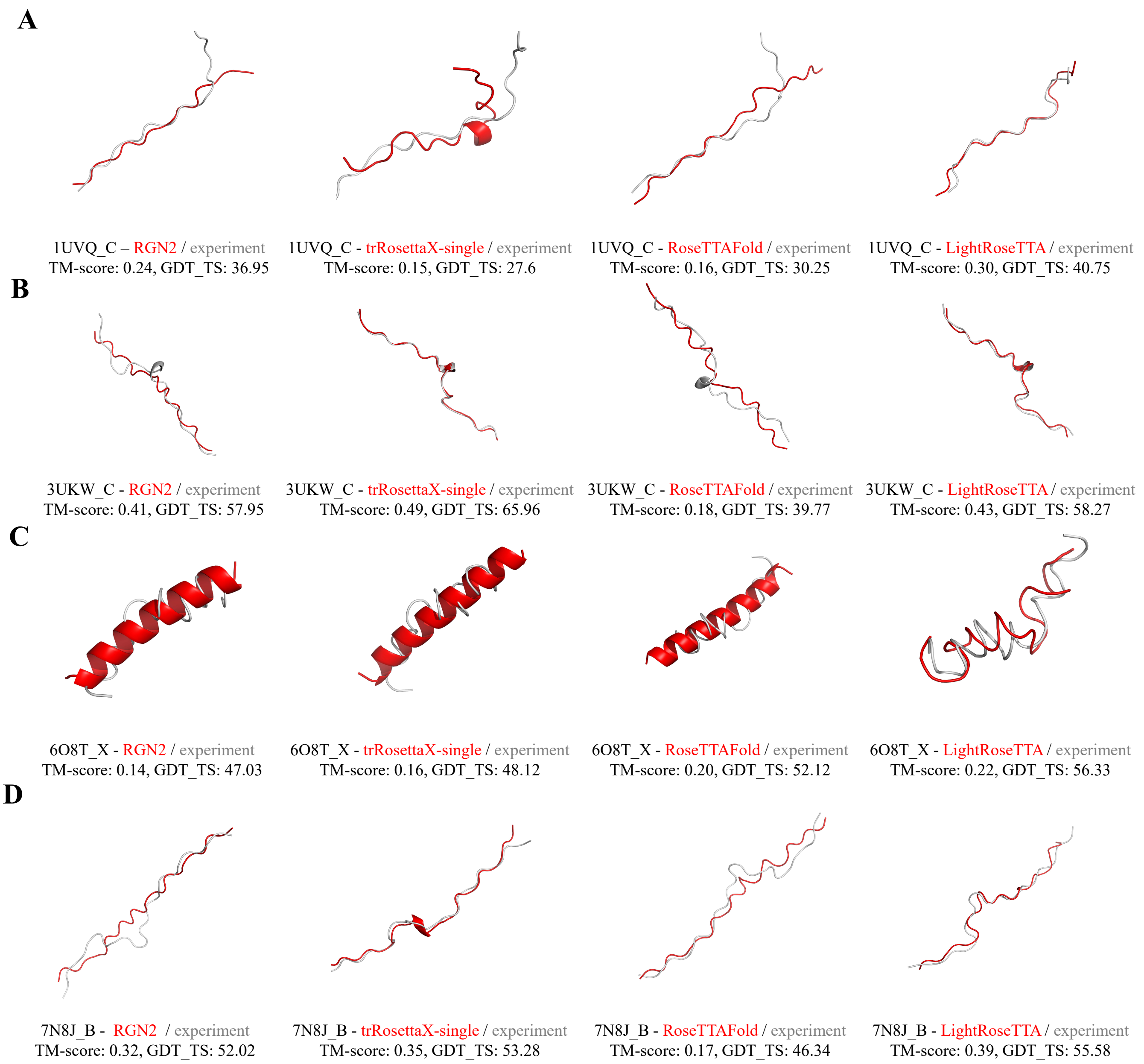

Figure S5: **The visualization of four methods' (RGN2, trRosettaX-single, RoseTTAFold, and LightRoseTTA) predicted protein structures with TM-score and GDT_TS on unseen proteins. A)** The prediction of Orphan target 1UVQ_C compared with the true (experimental) structure. **B)** The prediction of Orphan target 3UKW_C compared with the true (experimental) structure. **C)** The prediction of Denovo target 6O8T_X compared with the true (experimental) structure. **D)** The prediction of Denovo target 7N8J_B compared with the true (experimental) structure. *The prediction is colored red, and the true (experimental) structure is colored gray.*

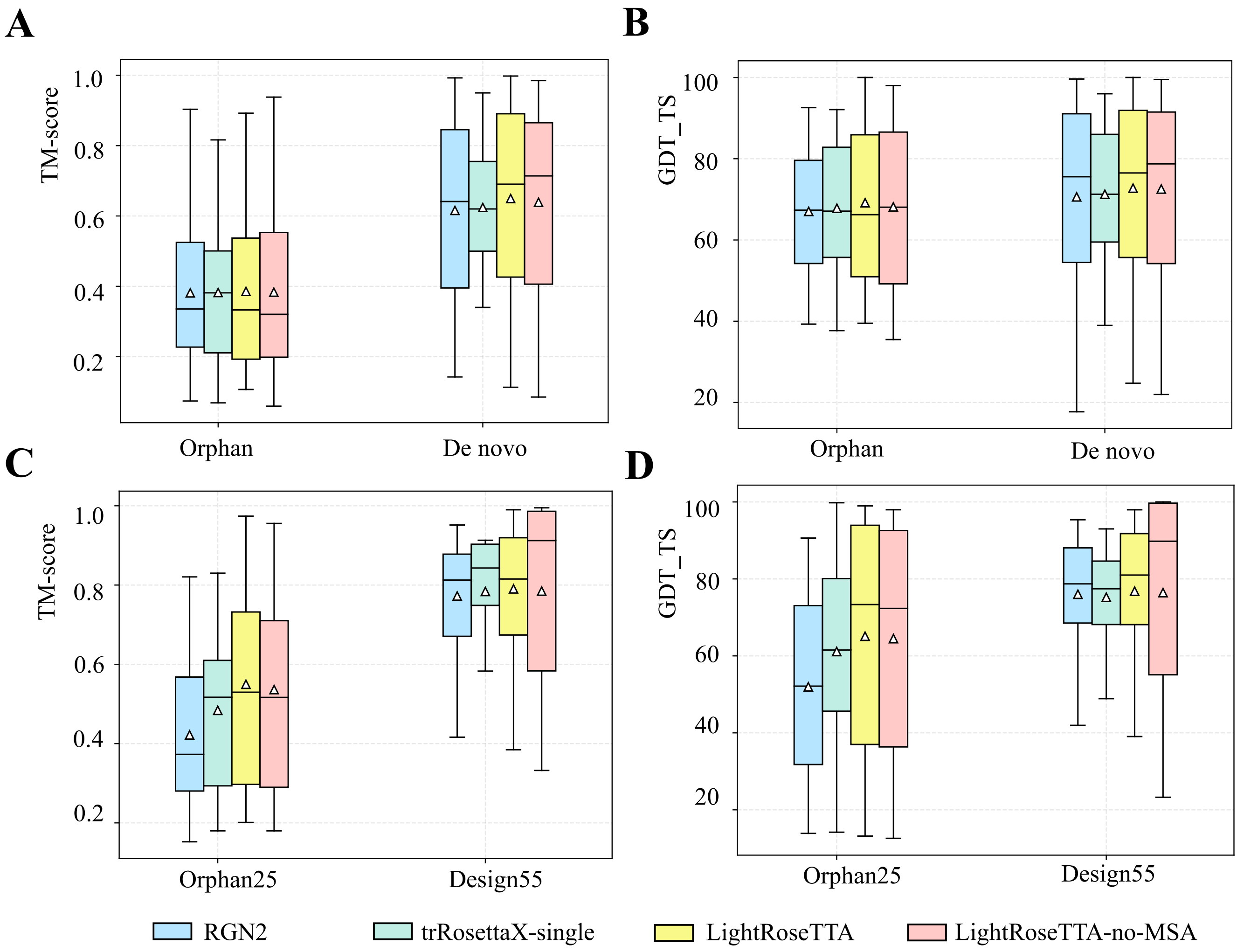

Figure S6: **The visualization of LightRoseTTA's prediction on protein datasets (Orphan, Denovo, Orphan25 and Design55) without MSA input. A)** The performance (TM-score) on the Orphan and De novo datasets. For each box in the figure, the center line, bottom line, and top line represent the median, first quartile, and third quartile, respectively. The horizontal lines along the top and bottom edges represent the maximum and minimum observations. **B)** The performance (GDT_TS) on the Orphan and De novo datasets. **C)** The performance (TM-score) on the Orphan25 and Design55 datasets. **D)** The performance (GDT_TS) on the Orphan25 and Design55 datasets.

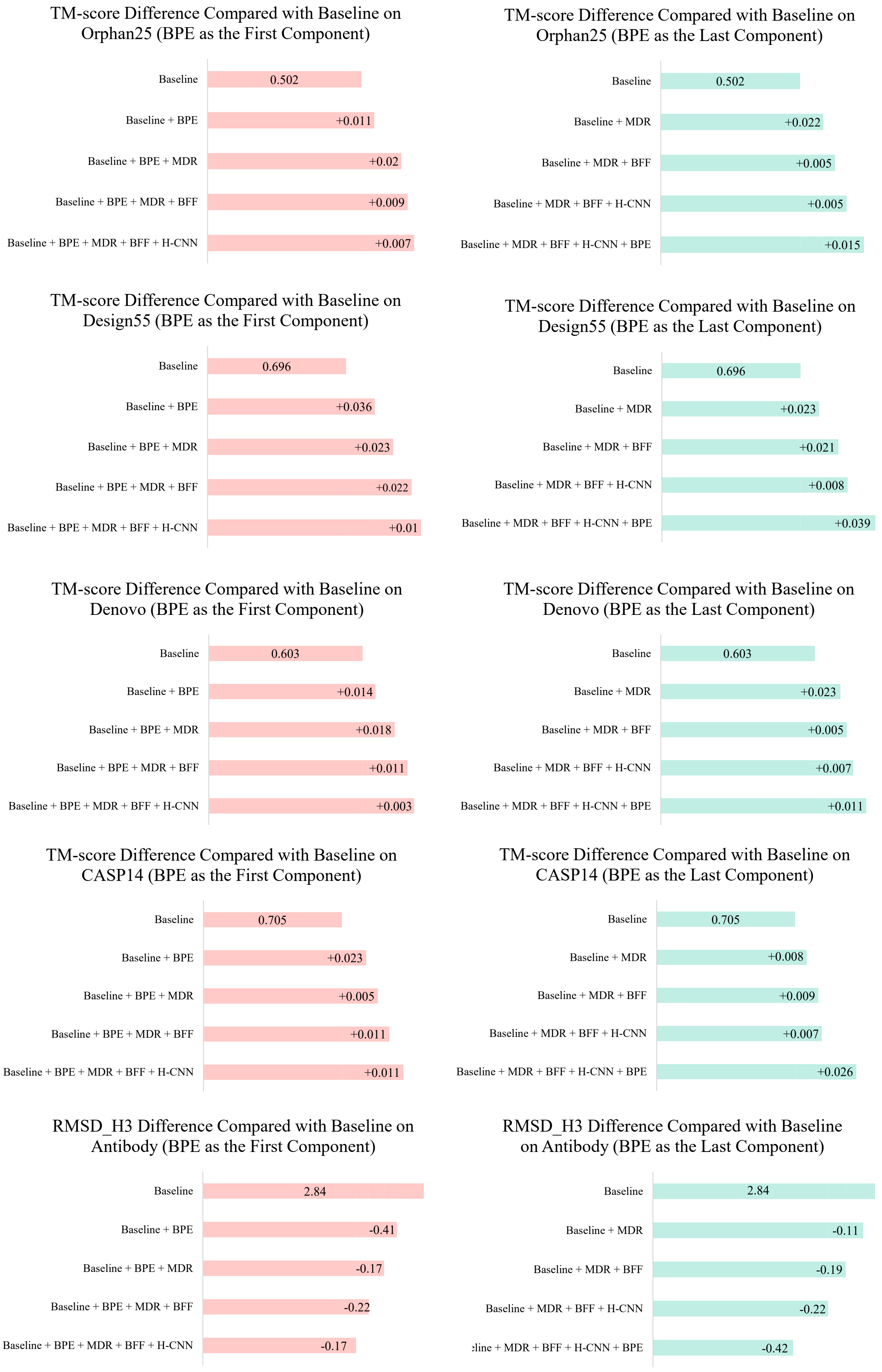

BPE: Backbone Potential Energy, MDR: MSA Dependency Reduction, BFF: Branch Feature Fusion, H-CNN: Hybrid CNN

Figure S7: **Ablation study on the Orphan25, Design55, Denovo, CASP14 and Antibody datasets. Specifically, we used the RMSD of CDR H3 as the performance metric for antibodies, so the smaller the prediction value, the better for the performance.**

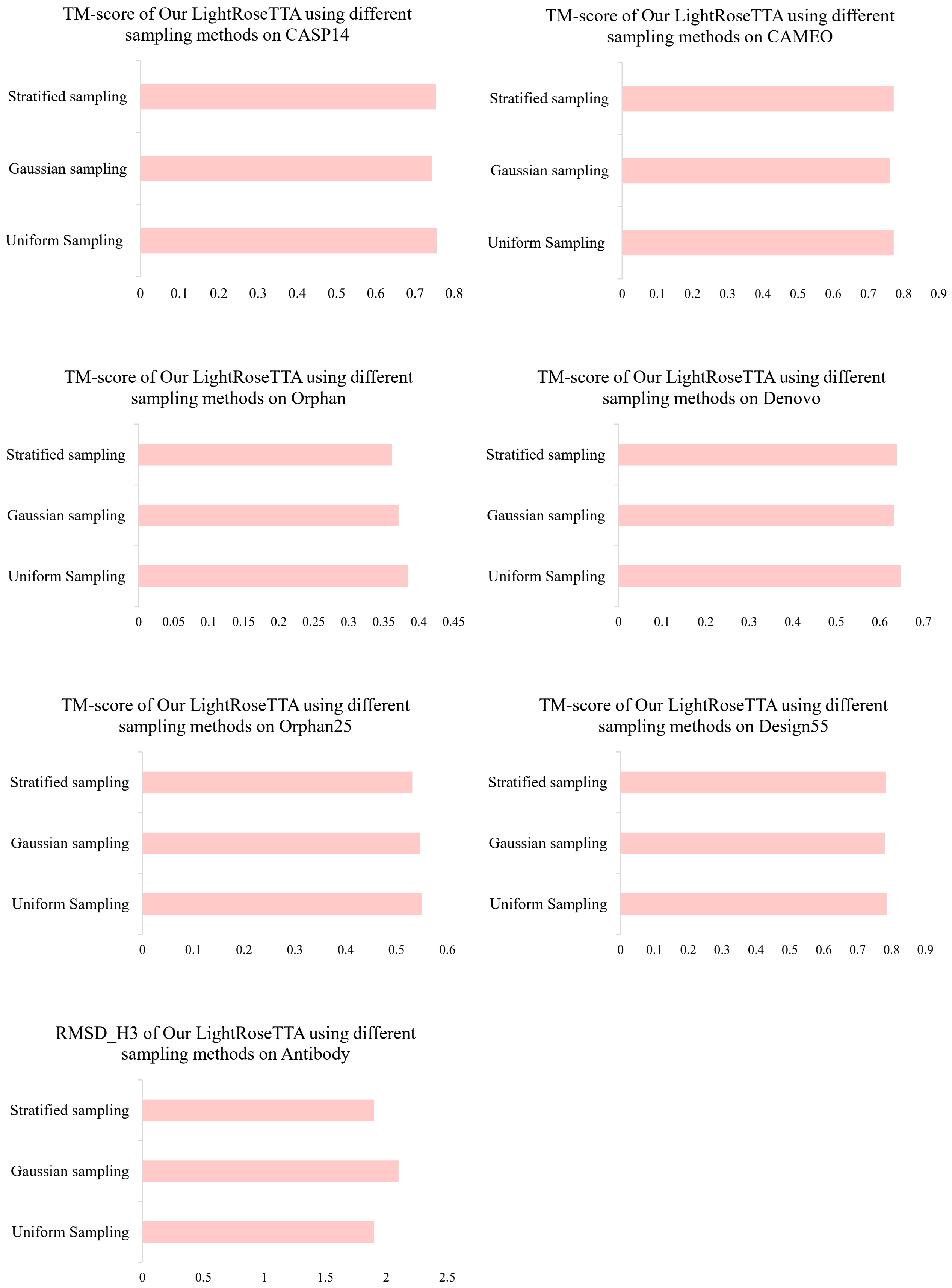

Figure S8: **Comparison of LightRoseTTA's performance with different sampling methods in MSA Dependency Reduction module on the CASP14, CAMEO, Orphan, Denovo, Orphan25, Design55 and Antibody datasets. Specifically, we used the RMSD of CDR H3 as the performance metric for antibodies, so the smaller the prediction value, the better for the performance.**

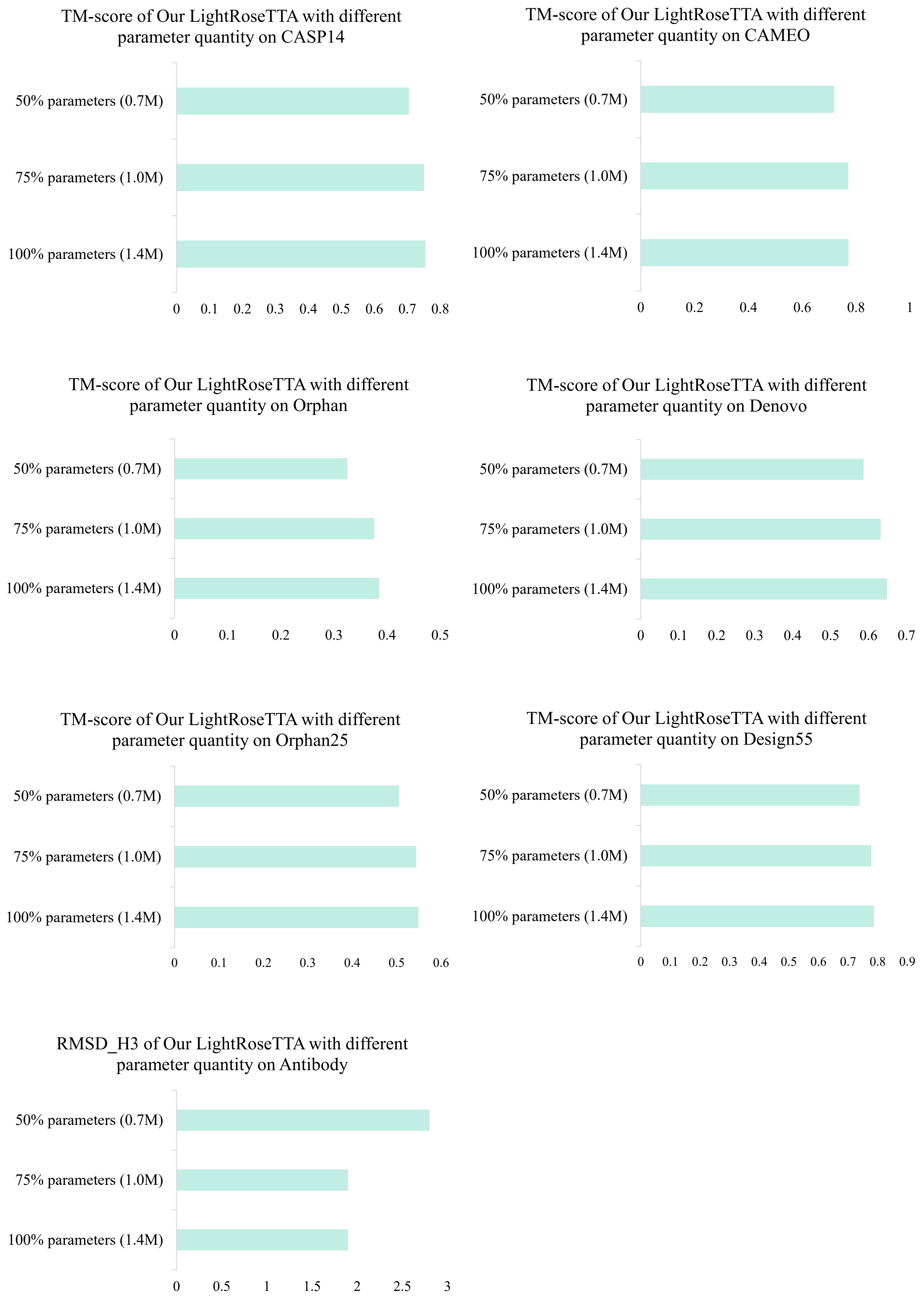

Figure S9: **Comparison of LightRoseTTA's performance with different parameter numbers on the CASP14, CAMEO, Orphan, Denovo, Orphan25, Design55 and Antibody datasets. Specifically, we used the RMSD of CDR H3 as the performance metric for antibodies, so the smaller the prediction value, the better for the performance.**

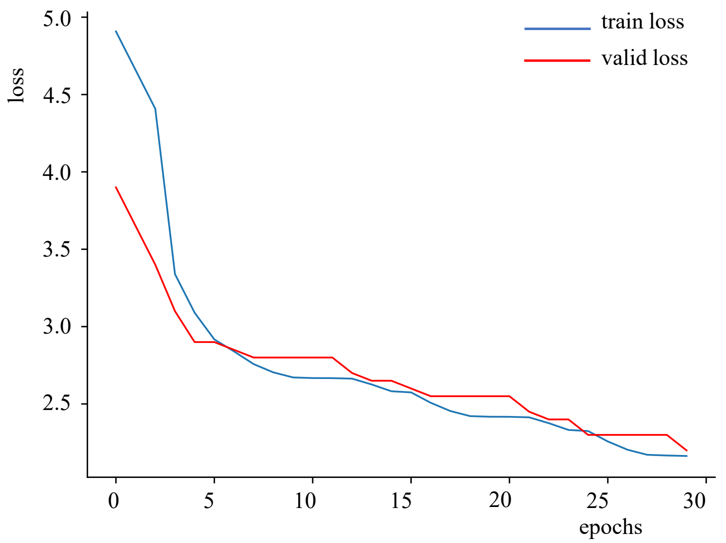

Figure S10: **The visualization of LightRoseTTA's training and validation loss curve.**

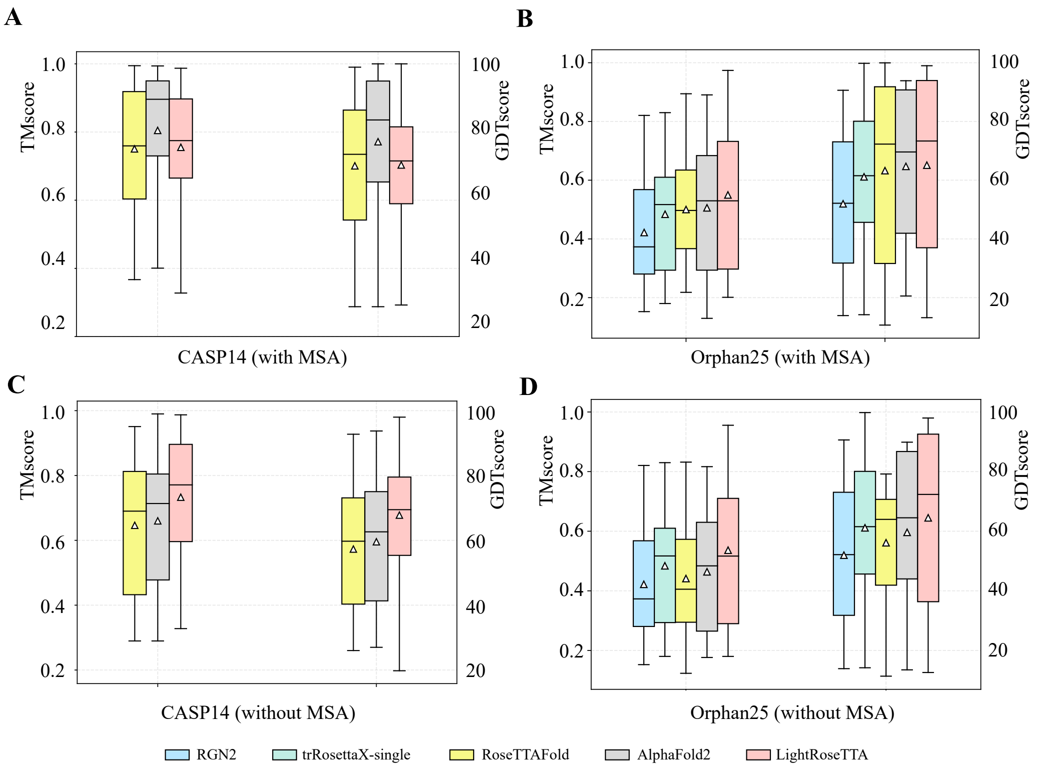

Figure S11: **The performance comparison on the CASP14 and Orphan25 dataset. Specifically, we add AlphaFold2 as a new comparison method. A)** The performance (TM-score and GDT_TS) on the CASP14 dataset (with MSA). For each box in the figure, the center line, bottom line, and top line represent the median, first quartile, and third quartile, respectively. **B)** The performance (TM-score and GDT_TS) on the Orphan25 dataset (with MSA). **C)** The performance (TM-score and GDT_TS) on the CASP14 dataset (without MSA). **D)** The performance (TM-score and GDT_TS) on the Orphan25 dataset (without MSA).

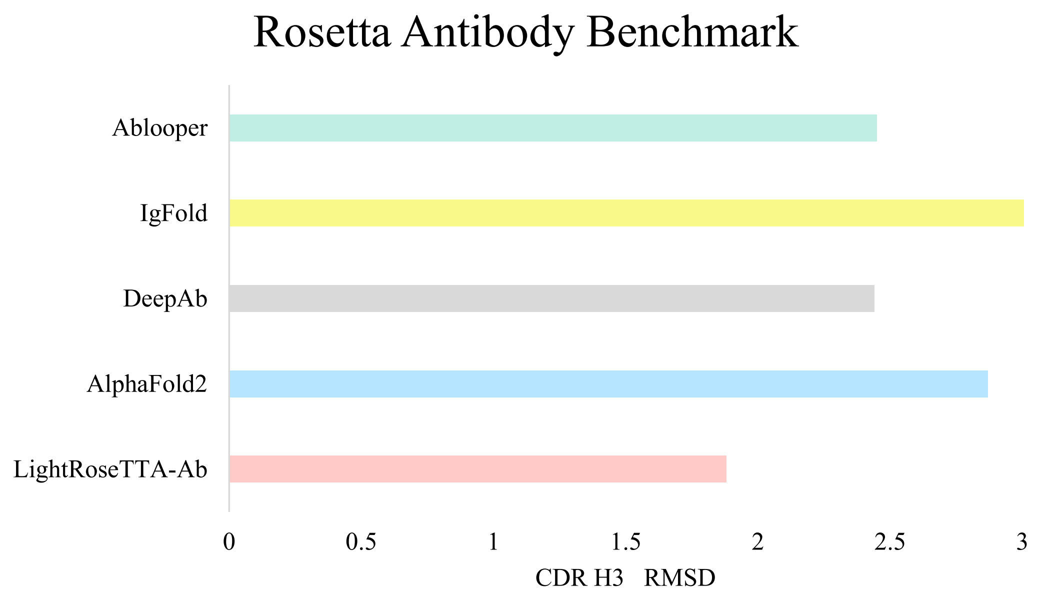

Figure S12: **The performance (RMSD-C_α of CDR H3) comparison on the Rosetta Antibody Benchmark dataset.**

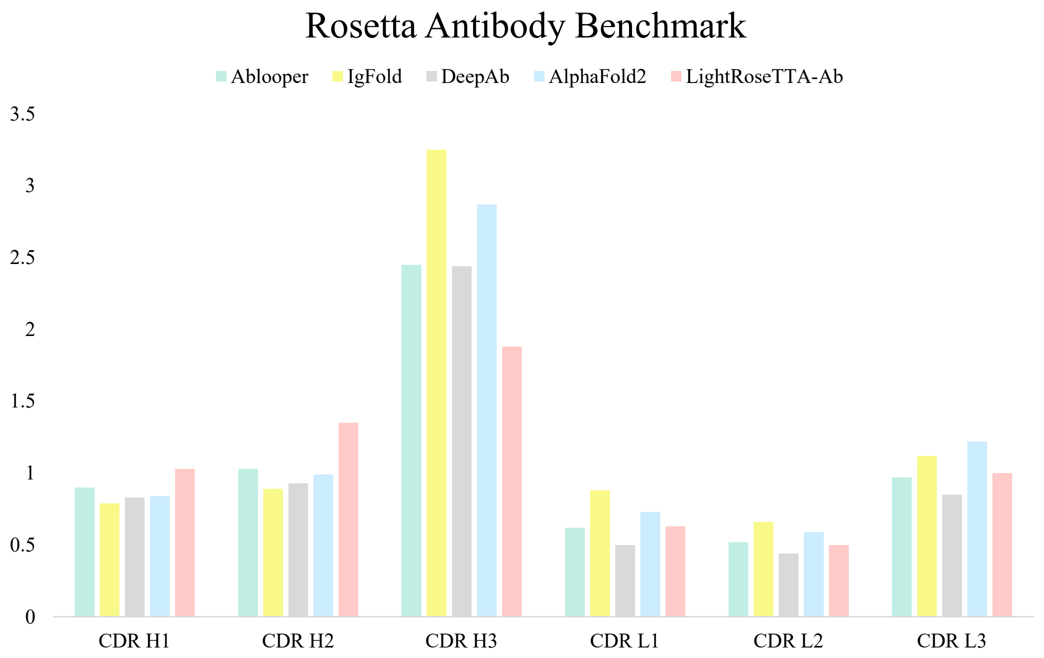

Figure S13: **The performance (RMSD-C_α of six CDRs) comparison on the Rosetta Antibody Benchmark dataset.**

References

[1] M. Baek, F. DiMaio, I. Anishchenko, J. Dauparas, S. Ovchinnikov, G. R. Lee, J. Wang, Q. Cong, L. N. Kinch, R. D. Schaeffer, et al., *Science.* **2021**, *117* 871.

[2] F. Ju, J. Zhu, B. Shao, L. Kong, T.-Y. Liu, W.-M. Zheng, D. Bu, *Nat Commun.* **2021**, *12* 2535.

[3] J. Yang, I. Anishchenko, H. Park, Z. Peng, S. Ovchinnikov, D. Baker, *Proc. Natl. Acad. Sci. U. S. A.* **2020**, *117* 1496–1503.

[4] R. Chowdhury, N. Bouatta, S. Biswas, C. Floristean, A. Kharkar, K. Roy, C. Rochereau, G. Ahdritz, J. Zhang, G. M. Church, et al., *Nat. Biotechnol* **2022**, *40* 1617–1623.

[5] W. Wang, Z. Peng, J. Yang, *Nature Computational Science* **2022**, *2* 804.

[6] J. A. Ruffolo, J. Sulam, J. J. Gray, *Patterns. 3*, 2.

[7] W. G. Touw, C. Baakman, J. Black, T. A. Te Beek, E. Krieger, R. P. Joosten, G. Vriend, *Nucleic acids research* **2015**, *43*, D1 D364.
